# Birdsong and the Neural Regulation of Positive Emotion

**DOI:** 10.3389/fpsyg.2022.903857

**Published:** 2022-06-22

**Authors:** Lauren V. Riters, Brandon J. Polzin, Alyse N. Maksimoski, Sharon A. Stevenson, Sarah J. Alger

**Affiliations:** ^1^Department of Integrative Biology, University of Wisconsin—Madison, Madison, WI, United States; ^2^Department of Biology, University of Wisconsin—Stevens Point, Stevens Point, WI, United States

**Keywords:** nucleus accumbens (NAc), emotion, positive affect, reward, motivation, songbird, communication, vocal behavior

## Abstract

Birds are not commonly admired for emotional expression, and when they are, the focus is typically on negative states; yet vocal behavior is considered a direct reflection of an individual’s emotional state. Given that over 4000 species of songbird produce learned, complex, context-specific vocalizations, we make the case that songbirds are conspicuously broadcasting distinct positive emotional states and that hearing songs can also induce positive states in other birds. Studies are reviewed that demonstrate that that the production of sexually motivated song reflects an emotional state of anticipatory reward-seeking (i.e., mate-seeking), while outside the mating context song in gregarious flocks reflects a state of intrinsic reward. Studies are also reviewed that demonstrate that hearing song induces states of positive anticipation and reward. This review brings together numerous studies that highlight a potentially important role for the songbird nucleus accumbens, a region nearly synonymous with reward in mammals, in positive emotional states that underlie singing behavior and responses to song. It is proposed that the nucleus accumbens is part of an evolutionarily conserved circuitry that contributes context-dependently to positive emotional states that motivate and reward singing behavior and responses to song. Neural mechanisms that underlie basic emotions appear to be conserved and similar across vertebrates. Thus, these findings in songbirds have the potential to provide insights into interventions that can restore positive social interactions disrupted by mental health disorders in humans.

## Introduction

Birds are admired for many qualities, including impressive migratory feats, complex singing behavior, and highly developed systems of mating and parental behavior; yet birds are not traditionally admired for being emotional. Darwin mentioned birds in only a few cases in *The Expression of the Emotions in Man and Animals* and in these cases focused on feather-ruffling and freezing responses as expressions of negative emotion (Darwin, 1897). More recent studies on avian emotion have also focused on negative states, such as fear or frustration (Papini et al., 2018), and some of the early research on the neural regulation of animal emotion was performed using distress calls in socially separated domestic chicks to assess negative affect (Panksepp et al., 1978, 1980; Vilberg et al., 1984). These studies demonstrate negative affective or fear states in birds; however, positive emotions are also necessary to elicit feeding, sexual, and social behaviors. These behaviors are essential for survival and reproductive success, but until recently the extent to which birds express positive emotions has been overlooked (Marino, 2017; Bertin et al., 2018).

There are many examples of birds engaging in behaviors that appear playful or pleasurable (Burghardt, 2005; Emery and Clayton, 2015; Pessoa et al., 2019). For example, there are reports of raptors dropping and catching objects, crows sledding, swans surfing, and parrots producing an emotionally contagious play call that is comparable to human laughter (Hewitt, 2013; Emery and Clayton, 2015; Schwing et al., 2017; Kaplan, 2020). Vocal behavior is considered a direct reflection of an individual’s emotional state (Cheng, 2003; Burgdorf et al., 2008; Panksepp, 2010; Briefer and Le Comber, 2012), which suggests that contrary to common perceptions, birds, and in particular songbirds, are among the most emotionally expressive species in the animal kingdom. When it comes to vocal production, few animals are as impressive, with approximately 4000 songbird species producing high rates of complex, species typical, learned songs. These songs convey information related to an individual’s motivational and emotional state and are designed to alter the emotional states of other birds.

Emotion is challenging to study from a neuroethological perspective. The term “emotion” is not consistently defined, we lack a common language around it, and it is often thought of as a completely internal experience. Indeed, conscious emotional feelings can only be confirmed in humans. However, emotions can be powerful motivators of behavior and emotional behaviors and responses can be quantified in non-human animals when conscious emotional feelings cannot. Here we define emotion as a mental state caused by neurophysiological changes associated with feelings. These include states of pleasure or reward and states of motivated reward seeking (Panksepp, 2011a), that are the focus of this review. Emotion, motivation, and reinforcement are tightly interrelated concepts that can be thought of as components of an integrated process that occurs when an animal faces a challenge or an opportunity. Emotion can occur in response to either innate or learned (i.e., conditioned) stimuli. Innate stimuli are intrinsically aversive or rewarding and can directly elicit emotional responses. Conditioned stimuli could serve as incentives (stimuli that motivate behavior) or reinforcers (stimuli that increase the probability that a learned behavior will occur) and can elicit emotional responses in anticipation of a challenge or an opportunity (LeDoux, 1996; Debiec et al., 2014). Thus, positive emotional states can occur in response to an intrinsic stimulus, can serve to bring an animal into proximity with a goal object, or can promote behaviors that increase opportunity.

Numerous studies on songbirds have provided fundamental insights into the neural regulation of vocal learning and production, but until recently emotion had not become an experimental variable in studies of birdsong (Cheng, 2003). In this review we make the case that birds are ostentatiously broadcasting positive emotional states and that the unique communication and social qualities of songbirds are ideal for providing fundamental insights into the neural regulation of positive emotional states. Many mental health disorders in humans, including depression, social anxiety, and autism spectrum disorder, are characterized by deficits in communication, and interactions that were once positive can become aversive leading to social withdrawal (APA, 2000; Pearlman-Avnion and Eviatar, 2002; Noens and van Berckelaer-Onnes, 2005; Knowland and Lim, 2018; Fox and Lobo, 2019; Gellner et al., 2021). Songbirds are one of only a few animals to produce complex, context-appropriate, learned vocalizations (Petkov and Jarvis, 2012; Vernes et al., 2021). Because neural mechanisms that underlie basic emotions appear to be conserved and similar across vertebrates (Panksepp, 2011a,b; Pessoa et al., 2019), findings in songbirds have the potential to provide insights into interventions that can restore positive social interactions disrupted by mental health disorders in humans.

## Birdsong as an Expression of an Anticipatory State of Reward Seeking

Songbirds sing in multiple contexts to attract and defend mates, repel rivals, to learn and practice songs, and in some cases for no immediate, obvious reason. There are two contexts in which song is strongly modulated by positive emotional states that will be the focus of this review. The first is the mating context. In seasonally breeding songbirds during the breeding season and in opportunistic breeders, males respond to the presentation of females with high rates of courtship song [e.g., (Jarvis et al., 1998; Riters et al., 2000)]. This song is critical for mate attraction. It reflects a male’s state of sexual motivation and is driven by the anticipation of female attraction and mating. The emotional state associated with sexually motivated song has been assessed using conditioned place preference (CPP) tests, which are commonly used to assess positive states in animals associated with feeding, drug use, sexual and playful behaviors (Carr et al., 1989; Tzschentke, 2007; Trezza et al., 2009). In brief, a bird is allowed to sing in an aviary and then immediately transferred to one of two distinct chambers separated by a divider. The following day the divider is removed and the amount of time a bird spends on either side of the apparatus is recorded. It is assumed that if the affective state associated with song is positive the bird will associate that positive state with the side of the chamber in which it is placed after singing and therefore spend most of its time in that chamber. This method was developed based on similar tests used to evaluate emotional states associated with sexual behavior in rodents (Agmo and Berenfeld, 1990; Tenk et al., 2009); its use in songbirds, interpretations and caveats have been discussed elsewhere in detail (Riters and Stevenson, 2012; Riters et al., 2014).

**FIGURE 1:**
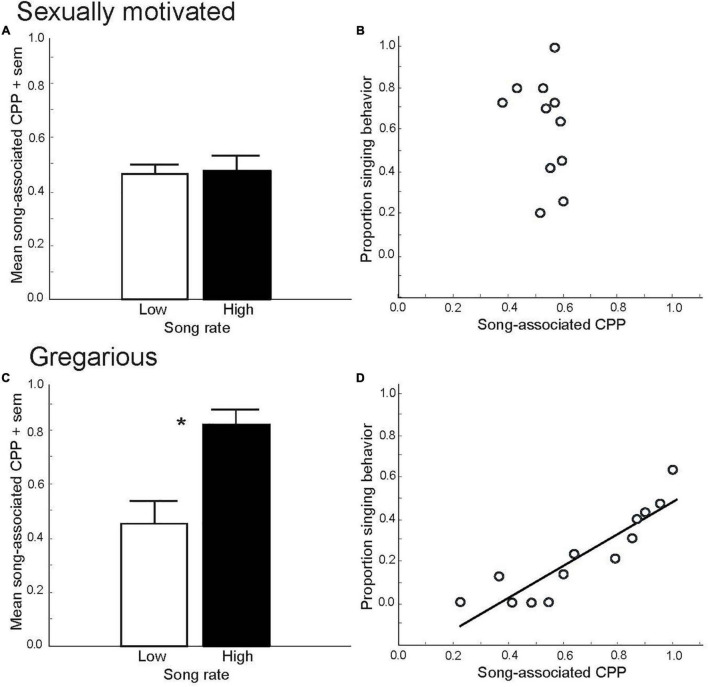
Conditioned place preference (CPP) tests demonstrate that sexually motivated and gregarious singing behavior reflect distinct emotional states. Mean proportion time spent in a location (i.e., song-associated CPP) that had been paired previously with either low (open bar) or high (filled bar) rates of singing behavior in male starlings singing in response to a female in spring **(A)** and singing in a flock in fall, *p = 0.006 **(C)**. Figures A and C replotted to show correlations between song and CPP in male starlings in spring, ns **(B)** and fall, *p* = 0.000002 **(D)**. *Y*-axis represents the proportion of all vocal behaviors that were songs produced by males during and just prior to being placed into a uniquely decorated place (i.e., a birdcage). The *X*-axis represents the proportion of time males later spent in that place the following day (CPP, considered a reflection of song-associated reward). Each point represents data from a single male. Figures redrawn from Riters and Stevenson (2012).

Although it is a common public perception that spring song is an expression of joy, experimental CPP tests suggest that it would be more accurate to interpret spring song as a reflection of a motivated, anticipatory state. This is supported by the finding that male European starlings, *Sturnus vulgaris*, do not develop preferences for places (i.e., CPPs) that had been paired previously with their own production of sexually motivated song (Riters and Stevenson, 2012) (Figures 1A,B). This indicates that the act of singing in this context is not itself an expression of a positive emotional state. Rather, males appear to sing in this context because they are seeking the pleasure induced by copulation. This idea is supported by numerous studies in birds that demonstrate copulation-induced place preferences (Domjan et al., 1992; Akins, 1998; Balthazart et al., 1998; Riters et al., 1998, 1999). Although males are well known for their singing behavior, there is a growing appreciation that females of many species also sing (Odom et al., 2014). For example, within a breeding context once a female pairs with a mate, female song may play a role in pair bonding, reproductive synchronization, extra-pair copulation or territorial defense and mate guarding (Sandell and Smith, 1997; Langmore, 1998; Collins, 2004; Catchpole and Slater, 2008). The affective states associated with song in these contexts has yet to be studied.

## Birdsong as an Expression of Intrinsic Positive Emotion

The second context in which song has been linked to a positive affective state is in a non-breeding context. Some seasonally breeding birds, such as starlings, form large mixed sex flocks outside their breeding seasons (Feare, 1984), and some opportunistically breeding birds, such as zebra finches, *Taeniopygia guttata*, spend much of their lives in large flocks (Zann, 1996; Goodson et al., 2012). While in these flocks, birds sing high rates of non-sexually motivated song. Song in this context is facilitated by the presence of flock mates (Gochfeld, 1977; Petrinovich, 1988; Jesse and Riebel, 2012), but unlike sexually motivated song it is commonly produced by birds facing away from other birds and it appears to be ignored (Feare, 1984; Zann, 1996; Riters et al., 2000). The immediate function of song in these flocks is not clear, but it is important for song learning and practice and proposed to play a role in flock cohesion (Riters et al., 2019). This is a type of song commonly referred to as undirected song in zebra finches (Dunn and Zann, 1996). Here we will refer to it as “gregarious” song when describing song in starling flocks and “undirected” when we discuss studies on zebra finches because, although these songs are produced in non-sexual contexts, they are not identical. Females of some species also sing in flocks. For example, the gregarious song described here is also produced by female starlings and studies reviewed below suggest that neural mechanisms underlying this type of song in males and females are shared (Stevenson et al., 2020; Maksimoski et al., 2021).

Although not part of his book on animal emotion, in *The Origin of Species*, Darwin proposed that although during the mating season male songs function mainly to attract females, outside the mating season males continue to sing for their own “amusement” (Darwin, 1871). Experimental CPP tests support this idea. Specifically, both male zebra finches and male and female starlings develop strong, positive preferences for places that were paired previously with their own production of gregarious or undirected song in flocks, and these preferences are linearly correlated with song rate (Riters and Stevenson, 2012; Riters et al., 2014; Hahn et al., 2017; Stevenson et al., 2020) (Figures 1C,D). These findings suggest that the act of singing in this context is tightly coupled to a positive emotional state, and it has been suggested that a natural function of the singing may be to strengthen group cohesion through a conditioned preference for a flock (Riters et al., 2019).

It has been proposed that song in gregarious contexts can be considered a form of rewarding play behavior similar to forms of play observed in multiple young animals as they practice sequences of motor events that are used later in adult reproductive contexts (Thorpe, 1956; Hartshorne, 1973; Ficken, 1977; Fagen, 1981; Burghardt, 2005; Riters et al., 2017). Performing behaviors for “amusement” (i.e., because they induce a positive emotional state) essentially defines play, and similar to other forms of playful behavior, gregarious song is facilitated by reunion with social partners, and it is initiated when an animal is fed, healthy and free from stress (e.g., in the absence of predators) (Panksepp and Beatty, 1980; Burghardt, 2005; Siviy et al., 2006; Yamahachi et al., 2020; Kim et al., 2021). This suggests that like other forms of play behavior, safety and the presence of flock mates may induce a positive emotional state that is conducive to singing, which may then be maintained by reward induced by the act of singing itself.

## Emotional State Is Reflected in Song Structure

Structural attributes of songs differ in association with emotional states. In numerous species, sexually motivated songs are found to be longer than non-breeding songs and to be more highly structurally and temporally stereotyped [e.g., (Sakata and Vehrencamp, 2012; Alger et al., 2016)]. Females prefer to mate with males that produce long, structurally stereotyped songs and males tend to avoid these males during competitions over breeding territories (Mountjoy and Lemon, 1991; Searcy and Yasukawa, 1996; Sakata and Vehrencamp, 2012). Downplaying these features in non-breeding contexts may function to promote social tolerance and group cohesion. The changes in structural features of song thus convey information to other birds about emotional state, and these vocal features can strongly influence the motivational state of others, as reviewed next.

## Birdsong as a Stimulus That Induces a Positive Emotional State in Others

Several studies demonstrate that hearing song can induce positive emotional states. For example, young male zebra finches readily learn to peck keys that trigger tutor song playback during the sensitive period for song learning, which demonstrates that hearing song alone can act as a reinforcer in a young male (Adret, 1993; Houx and Ten cate, 1999). Operant tasks that use song as a reinforcer also indicate that positive emotional states induced by hearing song play a role in adaptively shaping female mate choices. Female canaries, *Serinus canaria*, preferentially perform copulation solicitation displays in response to certain “sexy” male song syllables, and operant key peck tasks indicate that these songs are more reinforcing than songs that lack these syllables (Salvin et al., 2018). Female starlings trained to land on a perch-apparatus to play recorded male song spend more time on perches that play longer songs (Gentner and Hulse, 2000). These sexy elements appear costly to produce, suggesting that they accurately reflect male quality (Suthers et al., 2012). Thus, the positive emotional state induced by these elements adaptively shapes female mating choices by promoting preferential responses to high quality males. The fact that birds are willing to work (i.e., hop onto perches or peck keys) to hear song playback also indicates that hearing song, in combination with other morphological and behavioral traits, social and environmental context, history and individual differences (Kaplan, 2019; Fujii et al., 2022), may induce an anticipatory state of reward seeking in sexually motivated females. The positive emotional states induced by song likely in combination with these other variables also play a role in maintaining pair bonds in monogamous zebra finches, with operant responses to song reinforcement in females observed exclusively in response to a mate’s song (Tokarev et al., 2017; Coleman et al., 2019; Day et al., 2019).

**FIGURE 2:**
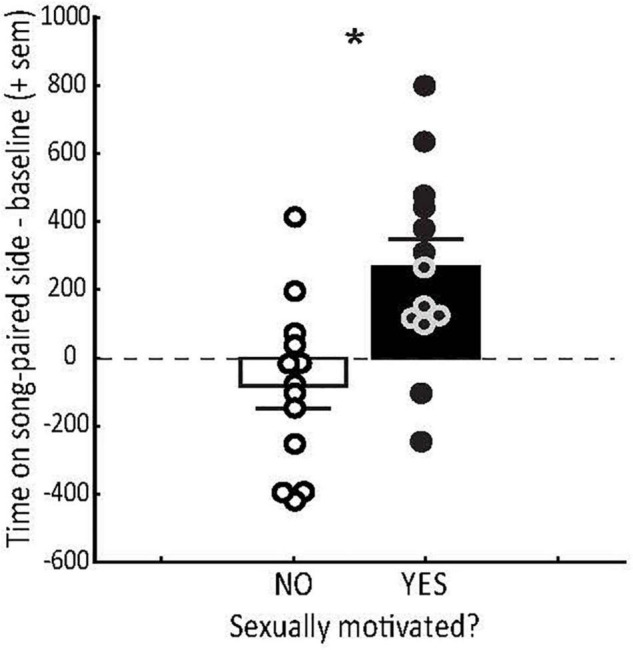
Conditioned place preference (CPP) tests demonstrate that hearing sexually motivated song induces a positive emotional state in sexually motivated females. Mean (+ sem) time spent (in the absence of song playback) on the side of the CPP apparatus previously paired with male starling courtship song (minus the baseline preference for that side during habitation) in female starlings that were not (open bar) or were (filled bar) sexually motivated as indicated by exploration of nesting sites. Each point represents data from a single female. Zero on the y axis indicates no preference. * = *p* < 0.0027. Figure redrawn from Riters et al. (2013).

The degree to which male song induces a positive emotional state in females can also depend on the reproductive state of the female. For example, female starlings that are sexually motivated and prepared to breed, as evidenced by active exploration of nesting sites, develop strong CPPs for places paired with playback of male courtship song (Riters et al., 2013) (Figure 2). Females that are not actively nesting do not develop similar place preferences (Figure 2). This suggests that the emotional state induced by hearing male song is plastic in adulthood and coordinated with female reproductive state such that song only induces a positive emotional state when females are prepared to breed. These studies demonstrate that song alone can serve as a reinforcer in sexually motivated females and that the emotional state induced by song plays a role in adaptive female mating decisions.

Emotional states induced by hearing gregarious song have not been well studied. To date, a single study in non-breeding condition female starlings demonstrated that on average females developed conditioned aversions to places that had been paired with playbacks of recordings of gregarious male song (Hahn et al., 2019). This was unexpected given the hypothesis that gregarious song-associated place preferences function to enhance flock cohesion. However, the prior CPP studies focused on the act of producing rather than hearing song [in females as well as males (Stevenson et al., 2020)]. It may thus be that the production of gregarious song in flocks is self-rewarding, but that hearing song alone is not. A form of vocal self-stimulation occurs in ring doves, in which a female’s own coos alter her endocrine physiology and behavior (Cheng, 2003). It may be that for birds in flocks, in females that sing (e.g., starlings), singing is also a form of self-stimulation and that producing song is more rewarding than hearing song. However, past studies also show that female starlings in overwintering flocks share song types with close neighbors (Hausberger et al., 1995). This raises the possibility that neighbor or familiar flock song may be more rewarding than the unfamiliar song stimuli that were used in the CPP study. The emotional valence of hearing flock songs requires additional research.

## A Role for the Nucleus Accumbens in Positive Emotion and Song Production

We make the case above that sexually motivated and gregarious singing behavior advertise and induce distinct emotional states. Studies in mammals indicate that basic emotions, including “seeking” and “play” (emotions associated with sexually motivated and gregarious song, respectively) are generated by subcortical structures that are considered highly evolutionarily conserved across vertebrates (Panksepp, 2011a,b). When it comes to positive emotional states the nucleus accumbens (NAc) is considered among the most important emotional pleasure centers in the brain (Berridge and Kringelbach, 2015), although it is important to note that NAc has many critical functions beyond reward (e.g., feeding, cognition, locomotion, and even the regulation of aversive responses) [reviewed in Floresco (2015)]. Here we focus on the role of NAc in reward-related processes.

An extensive body of literature demonstrates a role for NAc in both an anticipatory state of reward seeking and the experience of positive emotional states (e.g., playful states) in mammals (Berridge, 2007; Smith and Berridge, 2007; Trezza et al., 2011; Vanderschuren et al., 2016). Both dopamine and opioid neuromodulators act in NAc to modulate these emotional processes, with dopamine released into NAc from projections from the ventral tegmental area (VTA) implicated in anticipatory, reward seeking behaviors (Ikemoto and Panksepp, 1999; Berridge, 2007). This suggests a potential role for dopamine in NAc in male sexually motivated song production as well as female motivated responses to playbacks of male courtship song, reflected in operant responses, reviewed above.

In contrast to dopamine, opioids underlie the experience of an intrinsic positive or pleasurable emotional state, with activation of mu opioid receptors in specific “hedonic hotspots” in NAc found to induce reward in rats (Pecina et al., 2006; Berridge and Kringelbach, 2015). This suggests a potential role for opioids in NAc in the positive emotional state associated with gregarious or undirected singing behavior as well as the positive state induced by hearing male song as reflected in the finding that song playback alone can serve as an operant reinforcer and induce CPPs in females.

**FIGURE 3:**
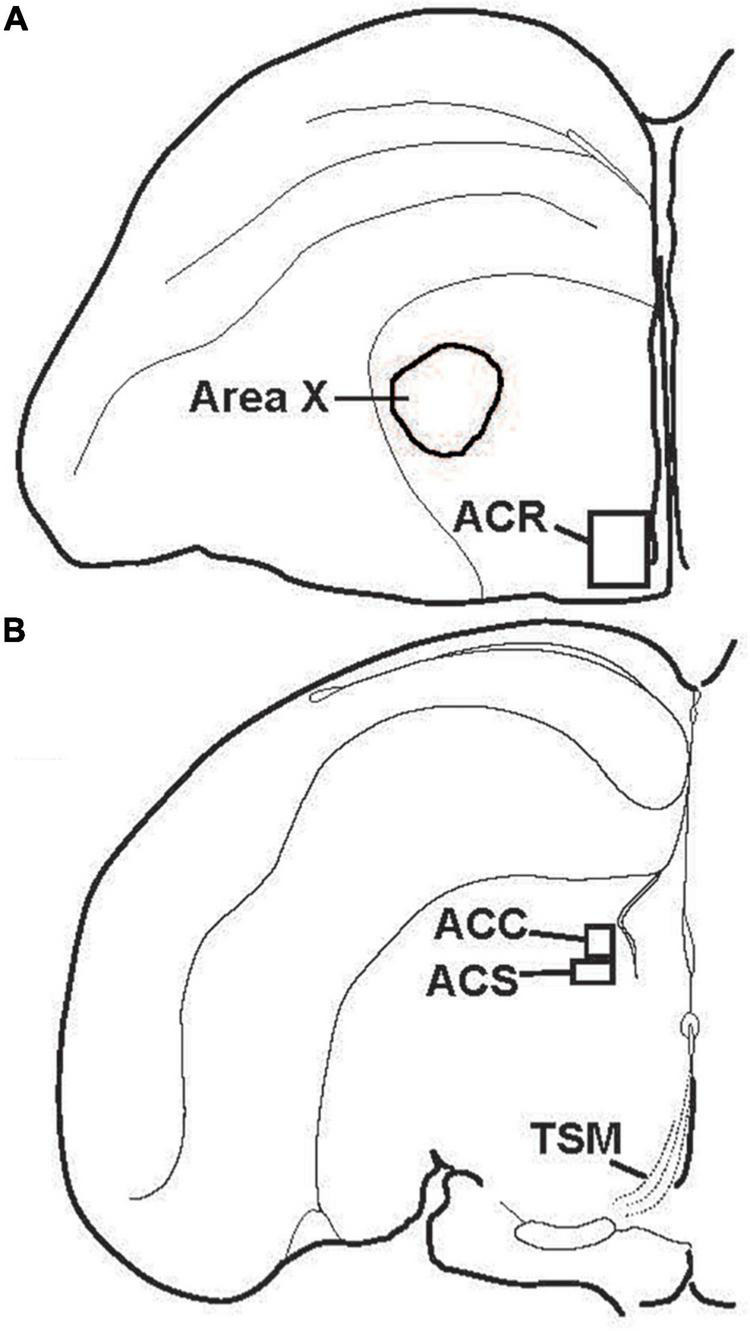
Location of subdivisions of the songbird NAc. Illustrations showing locations of **(A)** the rostral pole of the nucleus accumbens (ACR) and **(B)** the nucleus accumbens core (ACC) and shell (ACS) in the left hemisphere of coronal sections. TSM = tractus septomesencephalicus.

**FIGURE 4:**
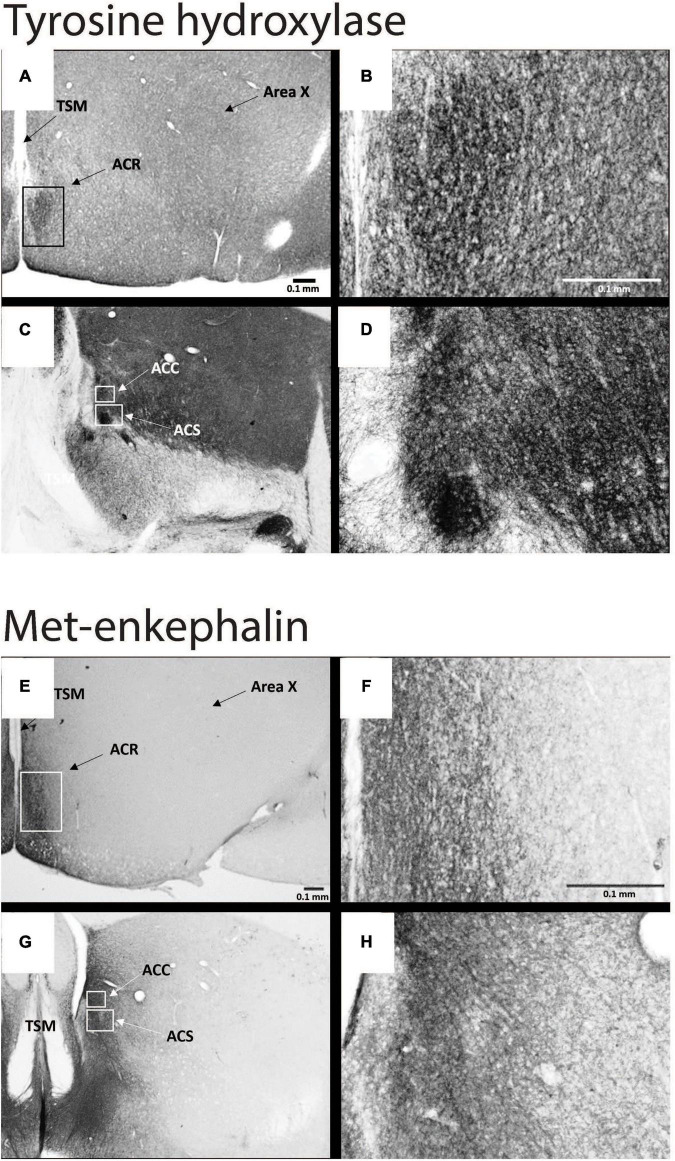
Subdivisions of NAc contain dopamine- and opioid-related immunolabeling in male starlings. Photomicrographs of immunolabeling for tyrosine hydroxylase at low **(A,C)** and high **(B,D)** magnification and of met-enkephalin at low **(E,G)** and high **(F,H)** magnification. See Figure 3 for abbreviations. Figures modified with permission from Polzin et al. (2021).

Despite over 40 years of research on the contribution of the NAc to positive emotional processes in mammals (Mogenson et al., 1979, 1980; Floresco, 2015), very little is known about the NAc in songbirds, perhaps because positive emotions in birds had not until recently become a serious research topic. Indeed, for many years the location of NAc in birds was in dispute, and in many atlases subregions of what is currently considered NAc were labeled as lobus parolfactorius or the lateral part of the bed nucleus of the stria terminalis (Karten and Hodos, 1967; Stokes et al., 1974; Youngren and Phillips, 1978; Kuenzel and Masson, 1988). In the early 2000s, NAc was newly delineated based on tract tracing and immunolabeling (Mezey and Csillag, 2002; Carrillo and Doupe, 2004; Reiner et al., 2004; Balint and Csillag, 2007; Balint et al., 2011; Husband and Shimizu, 2011; Gutierrez-Ibanez et al., 2016) (Figure 3), and recently in male starlings the rostral, shell, and core subdivisions of NAc were found to contain both dopamine- and opioid-related proteins, similar to mammals (Polzin et al., 2021) (Figure 4). These findings suggest potential functional homology between the avian and mammalian NAc and raise the possibility that dopamine and opioids in the songbird NAc may underlie emotional states of reward seeking and pleasure reflected in birdsong. Below we review studies that are beginning to provide support for this hypothesis and then integrate the NAc with a neurocircuitry that may provide a core, conserved system that generates positive emotional states reflected in birdsongs.

## Nucleus Accumbens and Birdsong as an Expression of an Anticipatory State of Reward Seeking

**FIGURE 5:**
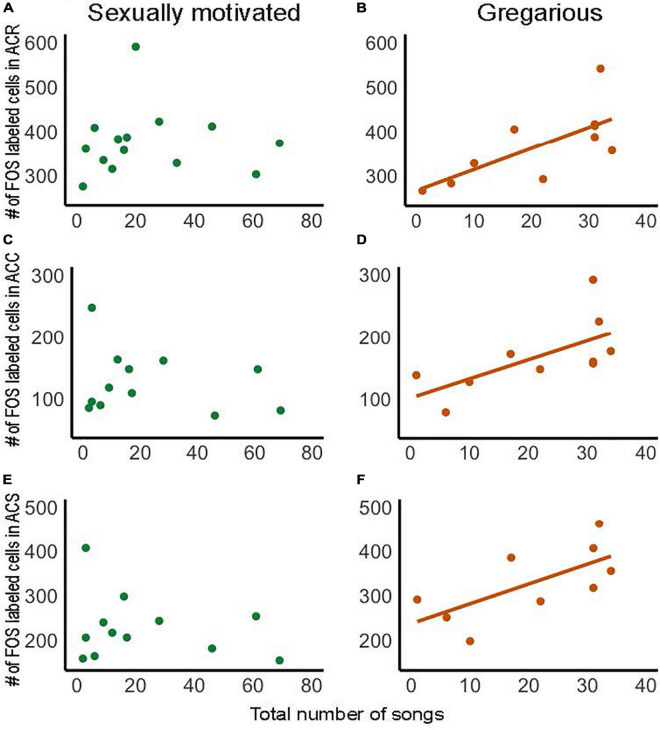
Numbers of FOS labeled cells within each subdivision of NAc correlate positively with the production of gregarious but not sexually motivated song in male starlings. Correlations in panel **(A,B)** show the rostral pole of the nucleus accumbens (ACR), **(C,D)** the accumbens core (ACC), and **(E,F)** the accumbens shell (ACS). Each point represents an individual bird. Presence of regression line indicates statistical significance [**(B)**: *R*^2^ = 0.49, *p* = 0.022; **(C)**: *R*^2^ = 0.42, *p* = 0.041, **(D)**: *R*^2^ = 0.46, *p* = 0.046]. Figure redrawn with permission from Polzin et al. (2021).

Dopamine in NAc is implicated in motivated, reward-directed behaviors, and several studies in rodents demonstrate that dopamine is released in NAc in males in response to the presentation of a sexually receptive female (Damsma et al., 1992; Wenkstern et al., 1993; Fiorino et al., 1997). This leads to the prediction that activity in NAc, and in particular dopamine release, may underlie anticipatory, seeking emotional states that motivate males to sing to attract females. Although the VTA, which is the major source of dopaminergic projections to NAc, is strongly implicated in song in this context (Yanagihara and Hessler, 2006; Hara et al., 2007; Heimovics and Riters, 2008; Nordeen et al., 2009; Kubikova and Kostal, 2010; Matheson and Sakata, 2015; Merullo et al., 2015), in a recent study no significant correlations were found between the production of sexually motivated song in male starlings presented with females and numbers of cells labeled for either the immediate early gene FOS or Egr-1 in NAc (Polzin et al., 2021) (Figures 5A,C,E). The absence of immediate early gene expression does not indicate the absence of neuronal activity, and not every immediate early gene is expressed in every brain area (Heimovics and Riters, 2005). Thus, these results certainly do not preclude a role for NAc in song in this context, which would be expected based on studies in rodents; however, this prediction must be tested in future research.

## Nucleus Accumbens and Birdsong as an Expression of Intrinsic Positive Emotion

Research on the role of NAc in song in non-sexual contexts is also in its early stages, but in a recent study in male starlings, numbers of cells labeled for the immediate early gene FOS in each subdivision of the NAc correlated positively with the production of gregarious song (Polzin et al., 2021) (Figures 5B,D,F). This contrasts with the absence of a correlation for sexually motivated song (Figures 5A,C,E), highlighting a potential role for NAc in gregarious song that differs from sexually motivated song. As reviewed above, unlike sexually motivated singing behavior, which is potentially rewarded later by mate attraction and copulation, gregarious singing behavior is positively coupled in a strongly linear fashion to an intrinsic positive emotional state (Figures 1C,D). It is thus possible that the linear positive relationships between gregarious, but not sexually motivated song, and immediate early gene activity in NAc reflect a difference in mechanisms that underlie song that reflects an ongoing intrinsic positive emotional state compared to a state of anticipatory reward seeking.

Song is clearly indicative of a highly motivated state within a mating context, yet birds must be motivated to initiate song in any context. Thus, given the role of dopamine in NAc in motivation, it would be expected that dopamine may play a role in initiating song in non-mating contexts as well. Consistent with this idea, there is evidence that dopamine increases in the striatal region area X in male zebra finches during undirected song, albeit to a lesser extent than during sexually motivated song (Sasaki et al., 2006). Moreover, a study in male zebra finches demonstrated that the latency to initiate undirected singing behavior (with latency considered a variable indicative of motivation) is inhibited by dopamine receptor antagonists (Kim et al., 2021).

**FIGURE 6:**
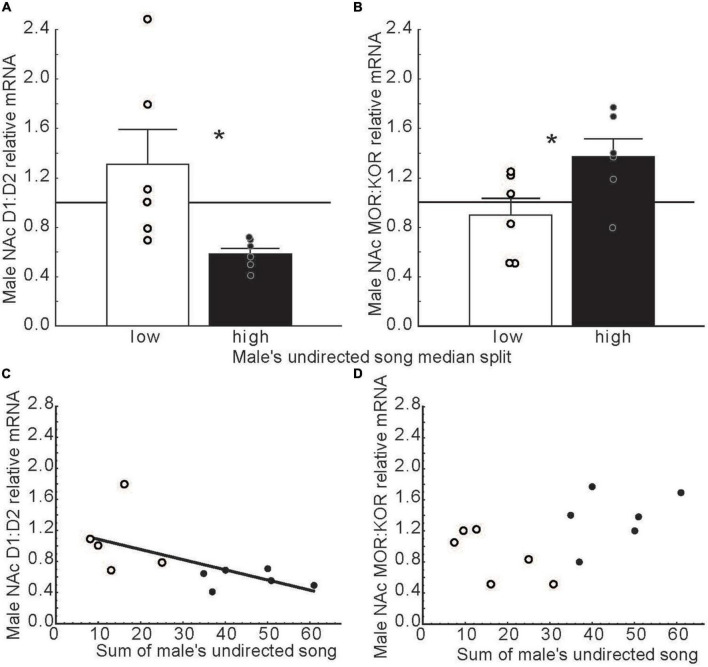
Dopamine and opioid receptor mRNA ratios in NAc differ in males that produce low versus high rates of undirected song. **(A,B)** Mean relative mRNA ratios + sem for D1:D2 and MOR:KOR in males that produce low (open bars) or high (filled bars) rates of song as determined using a median split. The point at which ratios diverge from 1:1 is indicated by a horizontal line. * = *p* < 0.05. **(C,D)** Scatterplots illustrating relationships between dopamine and opioid receptor mRNA ratios in NAc and undirected song in males. Each point represents an individual male. Open dots indicate low and filled dots represent high singers in bar graphs. The regression line indicates a significant correlation (*p* < 0.05). Data from Alger et al. (2021). Reproduced with permission.

Dopamine in NAc binds to receptors in the D1 and D2 families (Missale et al., 1998). These dopamine receptor subtypes activate opposing intracellular systems (Beaulieu and Gainetdinov, 2011; Soares-Cunha et al., 2016), and have different, sometimes opposing, impacts on behavior, including social behaviors [e.g., pair bond formation and maintenance in prairie voles, *Microtus ochrogaster* (Aragona et al., 2006; Aragona and Wang, 2007; Resendez et al., 2016; Lei et al., 2017)]. This suggests that ratios of D1 to D2 receptors in NAc may modulate motivational states. Consistent with this possibility, in long-term pair bonded zebra finches, ratios of D1 over D2 dopamine receptor expression in NAc related negatively to undirected song production (Alger et al., 2021) (Figures 6A,C). These findings potentially implicate dopamine in the intrinsic motivation to sing in any context and raise the possibility that D2 receptors in NAc play a predominant role intrinsically rewarding song production. D1 and D2 specific manipulations in NAc are now needed to test this possibility.

After dopamine in NAc initiates singing behavior, it may be that the ongoing positive emotional state that accompanies song in non-sexual contexts is maintained by the stimulation of opioid receptors in NAc. Opioids in NAc impact emotional states by binding to both mu and kappa receptors (Le Merrer et al., 2009). These receptors engage distinct second messenger systems (Belcheva et al., 2005) and have distinct effects on affective state, with mu opioid receptors generally inducing reward (Wise, 1989; Le Merrer et al., 2009) and kappa opioid receptors generally inducing negative affective states in mammals (Mucha and Herz, 1985; Shippenberg and Herz, 1986; Bals-Kubik et al., 1989), but see Castro and Berridge (2014). This suggests that ratios of mu over kappa receptors in NAc may also influence both an individual’s emotional state and singing behavior. Studies on male zebra finches support this possibility, with ratios of mu over kappa receptor expression related positively to male undirected song production (Alger et al., 2021) (Figures 6B,D). These findings suggest that a predominance of mu signaling in NAc may contribute to a positive emotional state associated with song in this non-sexual context. When combined with studies in mammals, these songbird studies suggest that dopamine, opioids, and receptor subtype ratios in NAc may fine tune emotional states to promote singing behavior in non-sexual social contexts.

**FIGURE 7:**
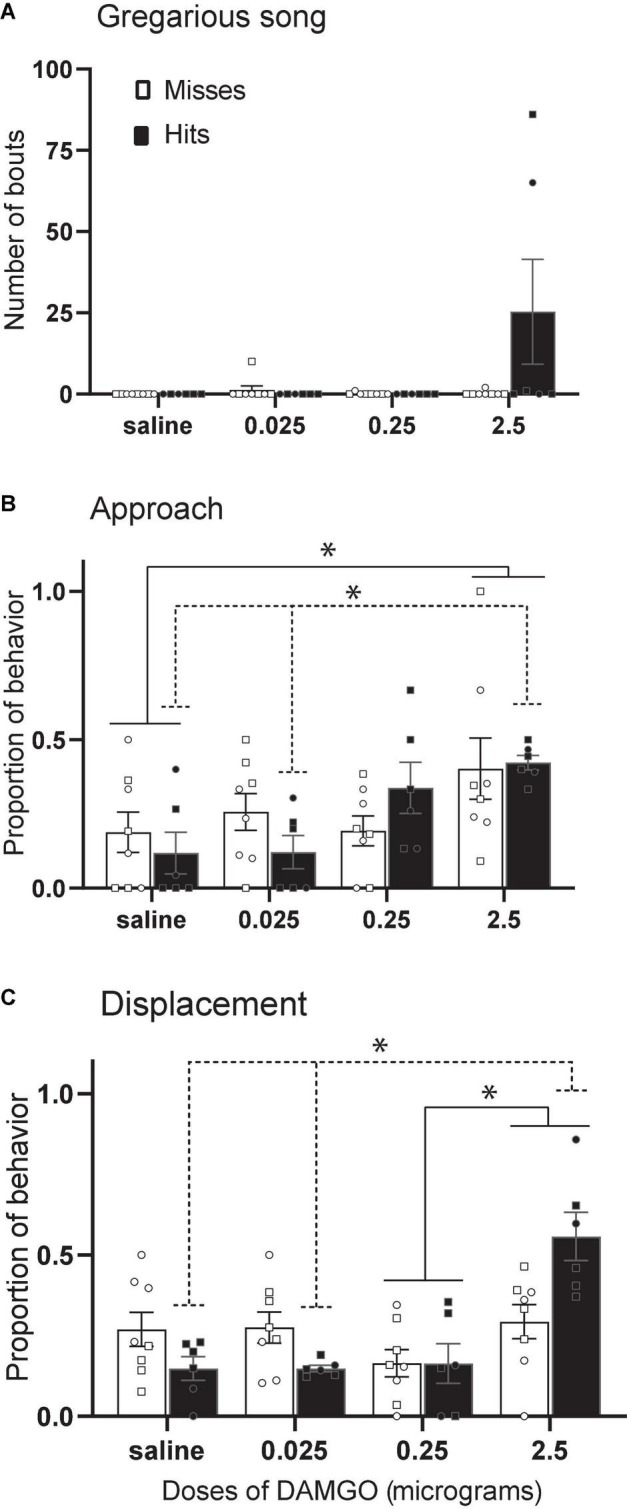
Stimulation of MOR in NAc increases gregarious song, approach, and displacements. Mean + sem **(A)** number of song bouts, **(B)** proportion of approach behaviors, and **(C)** proportion of displacements in male (squares) and female (circles) starlings in which the cannula tip missed the NAc (open bars; *n* = 8) or hit the NAc (filled bars; *n* = 6). Lines indicate significant effects (*p < 0.05). Misses were in numerous locations outside the NAc. Figure modified from Maksimoski et al. (2021).

The function of dopamine and opioid receptors and plasticity of the receptor subtype ratios in the avian NAc must be examined in future experiments, but progress has been made in the study of mu opioid receptors. In male and female starlings, the selective mu opioid receptor agonist fentanyl facilitates gregarious singing behavior (Stevenson et al., 2020), and in male zebra finches, peripheral injections of the non-selective opioid receptor antagonist naloxone suppress undirected song (Khurshid et al., 2010). A recent study suggests the NAc may be an important site of action. Infusion of the selective mu opioid receptor agonist DAMGO into the rostral NAc in male and female starlings stimulated gregarious singing behavior (Maksimoski et al., 2021) (Figure 7A). In addition, DAMGO dose-dependently increased both approach and displacement behaviors considered important for social spacing (Figures 7B,C). These results were interpreted to suggest a potential role for mu opioid receptors in the NAc in optimizing the pull of joining a flock with the push of potential agonistic encounters. Because the activation of mu receptors in NAc contributes to the reward value of non-sexual social behaviors in mammals [e.g., social play in young rats (Trezza et al., 2011)], it may be that DAMGO facilitates song and other behaviors important for flock cohesion by facilitating a positive emotional state.

## Nucleus Accumbens and Birdsong as a Stimulus That Induces a Positive Emotional State in Others

As introduced above, the fact that hearing song alone can function as a reinforcer in operant tasks indicates that hearing sexually motivated male songs can induce a positive emotional state. The fact that females are willing to work to hear these songs (reviewed above) also indicates that they can induce an emotional state of motivation or anticipation. This leads to the prediction that both dopamine (motivation/anticipation) and opioids (positive emotion) may be released into NAc in response to hearing male courtship song. A growing number of studies implicate NAc and dopamine in NAc in female motivated responses to these male songs. For example, in estradiol treated female white-throated sparrows, *Zonotrichia albicollis*, playbacks of male songs that induce female copulation solicitation also increase numbers of cells labeled for the immediate early gene Egr-1 in female NAc (Earp and Maney, 2012). In female zebra finches, dopamine transmission increases in rostral NAc selectively in response to the songs of a male mate, but not in response to non-mate songs (Svec et al., 2009; Tokarev et al., 2017). These findings are consistent with studies in female rats that demonstrate dopamine release in response to presentation of sexually active males (Pfaus et al., 1995), consistent with a conserved role for dopamine in NAc in the regulation of female motivated or anticipatory emotional responses to male songs designed to entice them to mate.

With respect to opioids, at least one study relates the opioid met-enkephalin in NAc to female responses to male courtship song. Specifically, in female starlings that are actively nesting (i.e., the females described above who found song to be rewarding in CPP tests), measures of immunolabeling for met-enkephalin in NAc shell and core combined tended to be higher than in females that were not nesting and did not find hearing song to be rewarding (Riters et al., 2013). These findings suggest a potential role for NAc in emotional states induced by male song. Research is now needed to test experimentally the working hypothesis that dopamine in NAc underlies an emotional state of anticipatory motivation while opioids underlie an emotional state of reward associated with responses to sexually motivated songs.

**FIGURE 8:**
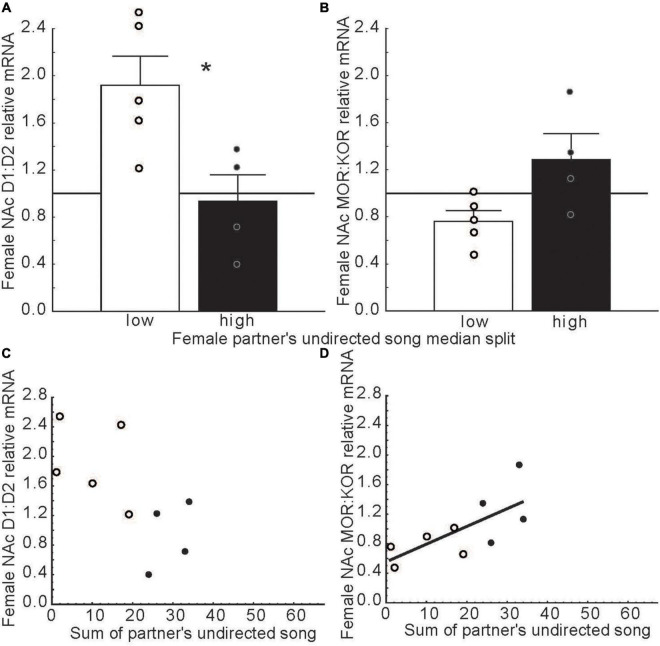
Dopamine and opioid receptor mRNA ratios in NAc differ in females whose partners produced low versus high rates of undirected song. **(A,B)** Mean relative mRNA ratios + sem for D1:D2 and MOR:KOR in females paired with males that produced low (open bars) or high (filled bars) rates of song as determined using a median split. The point at which ratios diverge from 1:1 is indicated by a horizontal line. **(C,D)** Scatterplots illustrating relationships between dopamine and opioid receptor mRNA ratios in the female NAc and the number of undirected songs produced by her partner. Each point represents an individual female. Open dots indicate females with male partners that sang at low and filled dots represent partners that sang at high rates in bar graphs. The regression line indicates a significant correlation (*p* < 0.05). Data from Alger et al. (2021). Reproduced with permission.

The role NAc in emotional states induced by hearing song outside the context of courtship has not been well studied. One study on this topic indicated that in long-term pair bonded zebra finches, females that were paired with males that produced high compared low rates of undirected song had lower ratios of D1 to D2 dopamine receptor expression and higher ratios of mu to kappa opioid receptor expression in rostral NAc (Alger et al., 2021) (Figure 8). The function of undirected song in this context is not clear, but it may play roles in maintaining the pair bond and in female reproductive investment (Silcox and Evans, 1982; Bolund et al., 2012). These findings raise the possibility that hearing undirected song may influence dopamine and opioid receptor expression in NAc to potentially impact female emotional responses to male song, which may reinforce pair bonds in monogamous species or promote flock cohesion in gregarious species. Additional research is needed on this topic.

## Nucleus Accumbens as Part of a Circuit Underlying Positive Emotions and Birdsong

The research reviewed here brings together for the first-time numerous studies that highlight a potentially important role for the songbird NAc in emotional states that underlie singing behavior. In this section we integrate this new information related to the NAc with prior research on other brain regions to develop a more comprehensive circuit that may infuse emotional state into vocal signals and responses to vocal signals. Much of the research on birdsong is focused on the “song control system”, a group of interconnected brain regions that is devoted to song learning, production, perception, and structural aspects of song (Nottebohm et al., 1976). These nuclei were commonly studied without reference to other neural systems, but in the late 1990’s studies began to focus on how a bird’s motivational state might be communicated to this system so that a bird would sing a structurally appropriate song in an appropriate context.

Because male courtship song is highly sexually motivated it was first proposed that brain regions involved in sexual motivation might directly or indirectly access the song control system to trigger the highly structurally stereotyped songs produced in a sexually motivated context. This led to a focus on the medial preoptic nucleus (POM), a region centrally involved in male sexual behavior across vertebrates (Dominguez and Hull, 2005; Balthazart and Ball, 2007). This region is well positioned neuroanatomically to influence the song control system (Riters and Alger, 2004), and several studies have now demonstrated an essential stimulatory role for the POM in the production of sexually motivated song in starlings and canaries (Riters and Ball, 1999; Alger and Riters, 2006; Alger et al., 2009, 2016; Alward et al., 2013, 2016).

The discovery of a role for POM in sexually motivated song was not surprising given its established role in male sexual motivation. However, it was somewhat surprising to find that the POM was also involved in song outside a sexually motivated context. For example, numerous studies implicate the POM in the production of gregarious song in male, as well as female, starlings (Alger and Riters, 2006; Riters, 2012; Kelm-Nelson and Riters, 2013; Riters et al., 2014; Stevenson et al., 2020), and CPP tests demonstrate the POM to play an important role in the positive emotional state that accompanies gregarious singing behavior (Riters et al., 2014; Stevenson et al., 2020). These studies demonstrate that the POM is a central node that adjusts singing behavior to reflect an individual’s motivational and emotional state (Alger and Riters, 2006; Riters and Stevenson, 2022).

**FIGURE 9:**
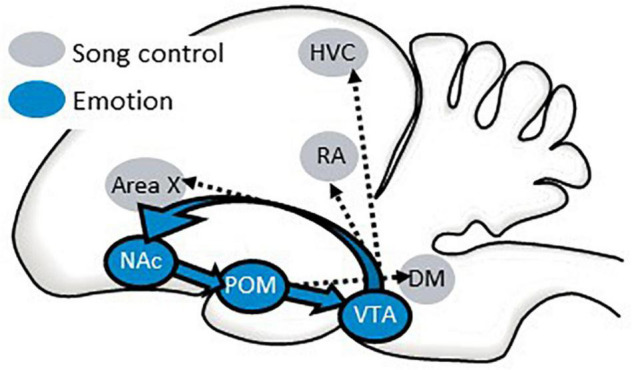
A possible pathway by which the NAc may coordinate an individual’s emotional state with singing behavior. Sagittal illustration of a songbird brain. A circuit from the preoptic area (POM) to the ventral tegmental area (VTA) to the nucleus accumbens (NAc) and back to the POM is proposed to regulate affective states that can influence singing behavior and perception through its projections to song control nuclei. See text for additional details.

One way in which the POM may infuse singing behavior with reward seeking and intrinsic positive emotion is by accessing the classic “mesolimbic reward system” through projections to the VTA which then sends projections to the NAc (Riters et al., 2019; Riters and Stevenson, 2022). There is evidence that the NAc also sends projections to the POM (Groenewegen and Russchen, 1984) forming a circuit that includes three areas strongly implicated in emotion and birdsong (Figure 9). This circuit directly accesses the song control system through projections from the VTA to vocal learning, perception, and production regions [HVC, robust nucleus of the arcopallium (RA), and area X] (Lewis et al., 1981; Appeltants et al., 2000, 2002) and from the POM to a song output region (DM) (Riters and Alger, 2004) (Figure 9). This creates a system by which an individual’s motivational state can be incorporated into song production and structure. Although this circuitry has not been as well studied in females as it has in males, a few studies implicate the POM in female emotional responses to hearing male song, reflected in CPP studies (Riters et al., 2013; Hahn et al., 2019). This raises the possibility that auditory inputs to this circuitry may also play a role in female emotional responses to male song.

## Additional Discussion and Remarks

The studies reviewed here demonstrate songbirds to be among the most emotionally expressive species in the animal kingdom, obtrusively advertising states of positive emotion and pleasure seeking. Because song is learned and complex, like human language and unlike vocalizations produced by rodents, songbirds can add layers to emotional communication that are not present in commonly studied rodent models. For example, in addition to emotional state, structural features of song communicate attributes of male health and past history to females (Nowicki et al., 2002; Peters et al., 2014); and recent studies demonstrate that songbirds can detect and generate prosodic vocalizations (Mol et al., 2017). For example, male zebra finches modify songs when singing to young birds during the period of song learning in a fashion similar to the way that humans alter vocalizations when speaking to young children (Chen et al., 2016). Moreover, gregarious singing in songbirds provides unique opportunities to understand learned vocal communication in relaxed, positive, non-sexual contexts, which are not observed in other commonly studied rodent or primate experimental systems.

We ended this review by highlighting a pathway from the POM to VTA to NAc that appears to contribute context-dependently to positive emotional states that motivate and reward singing behavior (as detailed in prior reviews, Riters, 2011, 2012; Riters et al., 2019; Riters and Stevenson, 2022). This pathway is also implicated in sexually motivated and playful behaviors in mammals (Trezza et al., 2011; Zhao et al., 2020), which underscores the idea that the bird brain has the machinery to regulate positive emotions and the similarity to mammals suggests that findings in birds may generalize to other vertebrates, including potentially humans. Rewarding, positive social interactions in humans are critical for mental health, yet there are few effective treatments for social behavior deficits in humans with mental health disorders. The studies reviewed here are beginning to fill a need for basic, mechanistic information on core emotional circuits that promote positive social interactions and may lay the groundwork for the development of novel treatments to restore positive social interactions in humans.

## Author Contributions

LR wrote the first draft of the manuscript. SA contributed to literature review and writing. BP, AM, and SA wrote sections of the manuscript and prepared the figures. All authors contributed to manuscript revision, read, and approved the submitted version.

## Conflict of Interest

The authors declare that the research was conducted in the absence of any commercial or financial relationships that could be construed as a potential conflict of interest.

## Publisher’s Note

All claims expressed in this article are solely those of the authors and do not necessarily represent those of their affiliated organizations, or those of the publisher, the editors and the reviewers. Any product that may be evaluated in this article, or claim that may be made by its manufacturer, is not guaranteed or endorsed by the publisher.
